# Household antimicrobial self-medication: a systematic review and meta-analysis of the burden, risk factors and outcomes in developing countries

**DOI:** 10.1186/s12889-015-2109-3

**Published:** 2015-08-01

**Authors:** Moses Ocan, Ekwaro A. Obuku, Freddie Bwanga, Dickens Akena, Sennono Richard, Jasper Ogwal-Okeng, Celestino Obua

**Affiliations:** Department of Pharmacology & Therapeutics, College of Health Sciences, Makerere University, P.O. Box 7072, Kampala, Uganda; Africa Centre for Systematic Reviews and Knowledge Translation, College of Health Sciences, Makerere University, P. O. Box 7072, Kampala, Uganda; Department of Microbiology, College of Health Sciences, Makerere University, P.O Box 7072, Kampala, Uganda; Department of Psychiatry, College of Health Sciences, Makerere University, P.O Box 7072, Kampala, Uganda; Infectious Disease Institute, College of Health Sciences, Makerere University, P.O Box 22418, Kampala, Uganda; Faculty of Epidemiology and Population Health, London School of Hygiene and Tropical Medicine, London, WC1E 7HT UK

## Abstract

**Background:**

Antimicrobial self-medication is common in most low and middle income countries (LMICs). However there has been no systematic review on non-prescription antimicrobial use in these settings. This review thus intended to establish the burden, risk factors and effects of antimicrobial self-medication in Low and Middle Income Countries.

**Methods:**

In 2012, we registered a systematic review protocol in PROSPERO (CRD42012002508). We searched PubMed, Medline, Scopus, and Embase databases using the following terms; “self-medication”, “non-prescription”, ‘self-treatment’, “antimicrobial”, “antimalarial”, “antibiotic”, “antibacterial” “2002-2012” and combining them using Boolean operators. We performed independent and duplicate screening and abstraction of study administrative data, prevalence, determinants, type of antimicrobial agent, source, disease conditions, inappropriate use, drug adverse events and clinical outcomes of antibiotic self-medication where possible. We performed a Random Effects Meta-analysis.

**Results:**

A total of thirty four (34) studies involving 31,340 participants were included in the review. The overall prevalence of antimicrobial self-medication was 38.8 % (95 % CI: 29.5-48.1). Most studies assessed non-prescription use of antibacterial (17/34: 50 %) and antimalarial (5/34: 14.7 %) agents. The common disease symptoms managed were, respiratory (50 %), fever (47 %) and gastrointestinal (45 %). The major sources of antimicrobials included, pharmacies (65.5 %), leftover drugs (50 %) and drug shops (37.5 %). Twelve (12) studies reported inappropriate drug use; not completing dose (6/12) and sharing of medicines (4/12). The main determinants of antimicrobial self-medication include, level of education, age, gender, past successful use, severity of illness and income. Reported negative outcomes of antimicrobial self-medication included, allergies (2/34: 5.9 %), lack of cure (4/34: 11.8 %) and causing death (2/34: 5.9 %). The commonly reported positive outcome was recovery from illness (4/34: 11.8 %).

**Conclusion:**

The prevalence of antimicrobial self-medication is high and varies in different communities as well as by social determinants of health and is frequently associated with inappropriate drug use.

**Electronic supplementary material:**

The online version of this article (doi:10.1186/s12889-015-2109-3) contains supplementary material, which is available to authorized users.

## Background

Self-medication refers to the use of medicines to treat self-diagnosed disorders without consulting a medical practitioner and without any medical supervision [1]. It is a common form of healthcare practiced in most parts of the world, with over 50 % of antibiotics purchased and used over-the-counter [2, 3]. Inadequacies in the healthcare delivery systems especially in resource limited countries such as inequitable distribution, high costs, inaccessibility, lack of health care professionals, unregulated distribution of medicines, patient attitudes towards physicians are some of the key drivers of self-medication [4, 5].

Non-prescription use of antimicrobial drugs is associated with the risk of inappropriate drug use which predisposes patients to drug interactions, masking symptoms of underlying disease and development of microbial resistance [4, 6–8]. The inappropriate drug use practices common in self-medication include; short duration of treatment, inadequate dose, sharing of medicines and stopping treatment upon improvement of disease symptoms [9]. Resistance to the available and affordable antimicrobial agents may further reduce the already limited therapeutic choices in treatment of common infectious diseases in developing countries, increasing the risk of morbidity and mortality [6].

In resource limited countries, the overall extent and determinants of self-medication with antimicrobial agents is difficult to quantify especially due to lack of monitoring and record keeping [10]. In addition the findings of studies on antimicrobial self-medication in these settings have not been consistent for example, a study by Yousef et al., (2008) [5] reported self-medication to be influenced by the high cost of health care, while lack of enforcement of legislations restricting over-the-counter sale of antibiotics was sighted as a reason for continued use of antimicrobial self-medication [11]. In addition, a recent global antimicrobial self-medication systematic review included few studies from developing countries, and excluded antimalarial self-medication [3]. Although various individual studies have examined antimicrobial self-medication in low and middle income countries, there has not been any systematic review done in this setting. There is need for evidence from well-designed studies on community use of antimicrobial drugs in these settings to help in planning and implementation of specific interventions on non-prescription antibiotic use.

We therefore performed a systematic review of observational studies to estimate the burden, risk factors and effects of antimicrobial self-medication in communities of low and middle income countries. Information on estimates of antimicrobial self-medication by geographical regions, sources of drugs, sources of drug information, clinical outcomes, antimicrobials agents and drug use practices are also summarized.

## Methods

### Protocol development

In 2012, we developed and registered a review protocol (#CRD42012002508) in the International Prospective Register of Systematic Reviews that is available at: http://www.crd.york.ac.uk/PROSPERO/. We observed the recommendations of the PRISMA statement [12] in developing this protocol and review conduct.

### Search strategy

RS, a librarian with Infectious disease institute Makerere University searched PubMed, Embase, Medline, and Scopus databases to identify studies that investigated antimicrobial self-medication in communities of low and middle income countries. The following search terms were combined using Boolean operators, antimicrobial, antibiotics, antibacterial, antimalarial, self-medication, non-prescription and the time period 2002-2012. Medical Subject Headings (MeSH) of the search terms was used in each case to maintain common terms across all data bases searched. All database searches were updated in February 2014. We searched bibliographies of included studies for additional articles. There was no language restrictions applied during the search neither did we search any grey literature for additional articles.

### Eligibility criteria for the articles included in the review

The review included studies on antimicrobial self-medication among adult individuals (18 years or older) in community households of low and middle income countries. We included only studies which did not assess chronic non-prescription antimicrobial use and reported the estimate of its prevalence. We excluded studies that assessed antimicrobial self-medication among; children, pregnant/breast feeding mothers, institutionalized elderly patients, students of health related courses and health professionals. In addition editorials, letters to the editor or comment publication type were also excluded. Studies done on children below eighteen years and institutionalized elderly were excluded from the review as they do not make independent treatment decisions; while students and workers of health profession are already exposed to unique knowledge and practices about antimicrobial use. Additionally, we excluded surveys of pharmacy bulk purchases or health facility exit interviews. This is because they do not provide reliable estimates of non-prescription antibiotic use despite being recommended by WHO for monitoring drug use [7]. We also excluded self-medication studies which measured knowledge only or attitude only or beliefs only and did not determine community behavior or practices. Studies on non-prescription antibiotic use done earlier than 2002 were excluded from the review as they are more likely to be affected by the regular changes in drug polices and treatment guidelines.

### Assessment of risk of bias of included studies

OC and EO independently assessed the risk of bias in the included studies and any disparities were resolved by discussion. We assessed the following potential sources of bias in observational studies using a tool of eight criteria that was adopted from the STROBE statement [13]. Selection bias due to sampling, selection bias due to responders or response rate (adequate if ≥ 60 %), detection bias due to recall (≤1 month; [14], detection bias due; social desirability, reliability of measurement tools and method of analysis used to assess factors associated with antimicrobial self-medication. We contacted authors of the included articles for clarification where information was missing. Each of the risk of bias criteria was assessed as low (scored as 0), moderate/high or unclear (scored as 1). We manually computed these scores into three levels of bias with 0-2 (low risk), 3-4 (moderate risk) and 5-8 (high risk).

### Data abstraction

We developed a data abstraction spreadsheet using Excel *version* 2007 (Microsoft Corporation, Redmond, Washington, USA). OC and EO conducted duplicate and independent abstraction of data from the included studies. We captured the following information, author, year of publication, journal, country where the study was done, recall period, study design, sample size, response rate, prevalence of antimicrobial self-medication, type of antimicrobial agents used, source of drugs, source of drug information, disease symptoms, determinants of antimicrobial self-medication, adverse effects, disease symptom resolution, risks associated with antimicrobial self-medication, reasons for self-medication, duration of drug use, and inappropriate drug use practices (not completing dose, sharing of drugs and short duration of use). In order to assess the determinants of antimicrobial self–medication, we considered data from only those studies that conducted multivariable regression.

### Data synthesis

We exported the Excel^©^ abstraction sheet to Stata^©^ software version 12.0 (Stata Corp, College Station, Texas, USA) for analysis. We performed both structured narrative and quantitative syntheses as appropriate. In the structured synthesis we generated descriptive summaries of the outcomes of interest from the included studies for the sources of drugs and drug information, types of antimicrobials, reported clinical outcomes, drug use practices and the determinants of self–medication. In order to estimate the prevalence of antimicrobial self–medication, we recomputed the primary study measures of proportions and the corresponding standard errors taking into regard the response rate.

We performed a DerSimonian–Laird Random Effects Meta–analysis to estimate the summary measure of the prevalence of antimicrobial self–medication, using the Stata^©^ command “metaan”. We displayed our findings in a forest plot and explored the high heterogeneity using the following sub – group analyses; region where the study was conducted (sub-Saharan Africa, Latin America, Middle East and Asia) as well as the levels of risk of bias (low, moderate and high) as shown in Table 2.

## Results

### Study selection

**Fig. 1:**
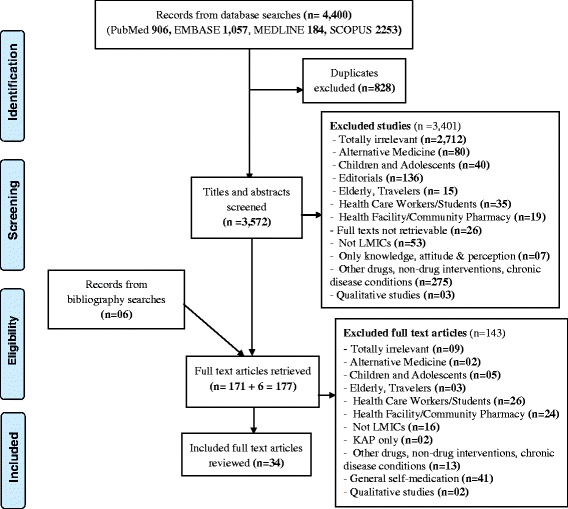
Flow diagram of study selection

The search of PubMed, Embase, Medline and Scopus data bases provided a total of 4,400 citations. After adjusting for duplicates 3,572 citations remained. Of these 3,401 studies were discarded since after reviewing their titles and abstracts, they did not meet the criteria. Seven studies were discarded as their full text was not available. The full text of the remaining 171 studies was reviewed in detail. A total of 143 studies did not meet the criteria and were discarded. Thirty four (34) studies met the inclusion criteria and were included in the systematic review. Additional six studies that met the criteria for inclusion were identified through searching the reference lists of located, relevant papers and searching for the studies that had cited these papers (Fig. 1). Two reviewers OM and EO screened the studies for inclusion and exclusion in the review with a kappa agreement of 0.74 (74 %).

### Characteristics of included studies

All the thirty four (34) studies finally selected for the review were cross-sectional observational studies published in English, French and Spanish and involved 31,340 participants. The recall period used in data collection varied among different studies, ranging from two weeks to one year (12 months). Most studies (19/34: 55.9 %) reported recall period used in data collection. Of the included studies, seven (7/34: 20.6 %) used a time lag of between two -to- four weeks [15–21] (Table 1). While three studies (3/34: 8.8 %) used twelve months [22–24].

**Table 1:** Summary of included studies on antimicrobial self-medication in LMICs

Study	Country	Recall period/Weeks	Prevalence of SM ( %)	Incorrect drug use	Outcomes	Disease symptoms treated
Abdo-Rabbo, 2003	Yemen	NR	4 %	NR	NR	Fever (4 %)
Agbor (2011)	Cameroon	NR	21.2 %		Symptoms resolved in a week (39.7 %)	Tooth ache (54.7 %), gingival bleeding (13 %)
Al-Azzam, 2007	Jordan	4	9.5 %	NR	NR	RTIs (39.1 %), GIT (4.9 %), UTIs (2.9 %), Ear (1.3 %)
Askarian, 2012	Iran	52	43.7 %	NR	NR	RTIs (73.1 %, GIT (41 %)
Auta, 2012	Nigeria	3	17.9 %	NR	NR	NR
Awad, 2005	Sudan	4	73.7 %	Inadequate dose (39 %), Short duration (39 %)	NR	RTIs (20.1 %), fever/malaria (5.5 %)
Bano, 2012	Pakistan	NR	55 %	Wrong dose, short duration	NR	RTIs (41.3 %), fever/malaria (80 %), GIT (61.3 %), Skin (72.6 %)
Barah, 2010	Syria	4	48.4 %	Stop taking drugs when symptoms improve (50 %)	Allergies (13 %), failed to cure (10 %)	NR
Chowdhury, 2009	Bangladesh	24	18.3 %	Stopped taking drugs when felt better (3.6 %)	Symptoms resolved (2.2 %)	Fever/malaria (55 %), GIT (9 %), Skin (11 %)
de Oliveira, 2004	Brazil	NR	10.1 %	Incorrect use (0.5 %)	Symptoms did not resolve (3.5 %)	NR
Deressa, 2003	Ethiopia	24	17.8 %	Dose not completed (4.3 %)	NR	Fever/malaria (97 %)
Enato, 2011	Nigeria	2	44.9 %	NR	Symptoms resolved (96 %)	Fever/malaria (57.6 %)
Hussain, 2011	Pakistan	12	17.8 %	NR	NR	RTIs (14.5 %), fever/malaria (57.6 %), GIT (8.4 %), Skin (8.6 %)
Jassim, 2010	Iraq	NR	63.5 %	Dose not completed (54 %), sharing drugs (12 %)	NR	RTIs (11.3 %), fever/malaria (8.1 %), GIT (11.3 %), Skin (4.9 %), UTIs (4.1 %), Ear (3.6 %)
Jombo, 2011	W. Africa	NR	38.4 %	NR	NR	NR
Lima, 2010	Brazil	NR	69.2 %	Sharing drugs	NR	RTIs, GIT
Mossa, 2012	Ethiopia	12	14.6 %	NR	NR	RTIs (14.1 %), fever/malaria (35.9 %), GIT (10.2 %)
Nounon, 2009	Argentina	NR	53.1 %	Stopped taking drugs when felt better (14 %)	Symptoms resolved (4 %)	RTIs (48 %), fever/malaria (18 %), Skin (1 %), UTIs (7 %)
Ngasha, 2011	Cameroon	NR	55.7 %	NR	Symptoms did not resolve	Fever/malaria
Okumura, 2002	Vietnam	NR	12.7 %	NR	NR	RTIs (3.1 %), GIT (1.6 %)
Onanuga, 2011	Nigeria	NR	45 %	Not completed dose (16.7 %), stopped taking drugs when symptoms resolved (28.3 %)	Experienced adverse effects (65.8 %)	NR
Onohwosafe, 2013	Nigeria	NR	54.8 %	NR	NR	Fever/malaria (49.6 %)
Osemene, 2012	Nigeria	NR	91.4 %	NR	NR	RTIs (15.3 %), fever/malaria (5.5 %), GIT (10.2 %), UTIs (43.5 %), Ear (2 %)
Oyetunde, 2010	Nigeria	NR	25.9 %	Short duration (90 %)	NR	NR
Omole, 2010	Nigeria	12	35.7 %	NR	Not cured (33.7 %), cured (12.9 %)	Fever/malaria, GIT
Sanjana, 2006	Indonesia	52	42 %	NR	Adverse effects (23 %), relapse of malaria	Fever/malaria (93 %), GIT (17 %)
Sapkota, 2010	Nigeria	12	25.2 %	NR	NR	None specific symptoms
Sarahroodi, 2009	Iran	12	54.5 %	Not completing dose (74.2 %)	NR	RTIs (66.7 %), GIT (23 %)
Sawalha, 2008	Palestine	24	19.4 %	Not completing dose (59.9 %)	NR	RTIs (30.3 %), Ear (3.3 %)
Shankar, 2002	Nepal	24	59.2 %	NR	NR	Fever/malaria
Shehadeh, 2012	Jordan	52	30 %	Not completing dose (38.5 %)	ADRs (69.6 %), Allergy, harm the teeth	RTIs (31 %), UTIs (4.7 %), Ear (3.8 %)
Sihavong, 2006	LPR	52	91 %	Short duration of taking drugs (79 %)	NR	UTIs (78 %)
Widayati, 2011	Indonesia	4	8.1 %	Short duration of taking drugs (36.6 %)	NR	RTIs (31.8 %), fever/malaria (12.2 %)
Yousif, 2002	Sudan	NR	46.9 %	Sharing drugs (59.3 %), Not completing dose (28.8 %)	NR	NR

LMICs: Low and Middle Income countries, NR: Not Reported, RTIs: Respiratory Tract Infections, GIT: Gastrointestinal tract, UTIs: Urinary Tract Infections, LPR: Lao People’s Republic

Of the 34 studies, only 7 (20.6 %) established determinants of antimicrobial self-medication using multiple regression analysis. Fifteen studies (15) were from Sub-Saharan Africa, eight (08) Asia, eight (08) Middle East and three (3) from South America. Nineteen (19) studies reported the recall period used during data collection. Less than one month [15–21], 2-5 months [25–29] and 6-12 months [22–24, 30–33] while fifteen studies did not report the recall period use [34–48] (Table 1).

The majority of studies, 79.1 % (27/34) reported symptoms related to infections of; respiratory tract, gastrointestinal system, eye, ear, urinary system, skin and malaria as the reason for self-medication. Of the thirty four (34) studies, five (5) reported the duration of treatment using antimicrobial self-medication in management of the illness. Four-to-seven days [21] (antibiotics); one-to-three days [32] (antimalarial); three days [23] (antimalarial); and three-four days [16, 17] (antibiotics).

### Risk of bias in the included studies

The majority of included studies (27/34: 79.4 %) did not assess determinants of antimicrobial self-medication using multivariable regression analysis. Fourteen of the included studies (14/34: 41.1 %) had low risk of bias, twelve (12/34: 35.3 %) had moderate risk of bias while eight (8/34: 23.5 %) had a high risk of bias.

Following the risk of bias assessment criteria used, most of the included studies had potential risk of; bias due to method of analysis used in establishing associated factors (28/34: 82.4 %), recall bias (23/30: 67.6 %), selection bias (12/34: 35.3 %), detection bias due to social desirability (13/34: 38.2 %), selection bias due to baseline characteristics (17/34: 50 %) and selection bias due to sampling criteria (13/34: 38.2 %) (Additional file 1: Appendix 1).

### Antimicrobial medicines commonly used in self-medication

The major categories of antimicrobial agents reportedly used in self-medication included antimalarial and antibacterial. Of the thirty four studies included in the review, seventeen (50 %) investigated only antibacterial drugs, eight (23.5 %) antibacterial and antimalarial drugs, five (14.7 %) antimalarial drugs only, and four (11.8 %) studied multiple antimicrobial agents used in self-medication. The antimalarial medicines commonly used in self-medication included, chloroquine, sulfadoxine-pyrimethamine, halofantrine, Artemether-Lumefantrine, and quinine. While the antibacterial agents used included; ampicillin, tetracycline, penicillin, metronidazole, ceftriaxone, kanamycin, ciprofloxacin, amoxicillin, fradiomisin-gramisidin, norfloxacin and doxycycline (Table 2).

**Table 2:** Characteristics of antimicrobial drugs used in self-medication

Type of antimicrobial	Class of antimicrobial	Drug source	Source of information
Antibacterial	B-lactam, Tetracycline, fluoroquinolone, macrolide, quinolone, aminoglycoside, others	leftover, pharmacy, drug shop, friends/relatives	drug seller, self, drug leaflet, past prescription, friends
Antimalarial	Artemisinins, 4-aminoquinolines, 8-aminoquinlines Cinchona alkaloid Sulfonamides/sulfone Diaminopyrimidine	pharmacy, leftover, friends/relatives	past prescriptions, self, drug seller, friends
Antifungal	Azoles	pharmacy, leftover	drug seller, self
Anthelmintic	Imidazole	pharmacy, leftover	drug seller, self, past prescription, friends

### Source of medicines, information and benefits of antibiotic self-medication

Information on antimicrobial agents used in self-medication in developing countries is obtained from various sources. Majority of the studies reported drug sellers or pharmacists and relatives or friends. The other reported sources include; past successful use and drug leaflets. The antimicrobial drugs used in self-medication were obtained from various sources such as pharmacies, leftover drugs, hospitals, gifts from friends and drug shops.

The antimicrobial agents used in self-medication were obtained from various sources such as pharmacies, 61.8 % (21/34); leftover drugs, 41.2 % (14/34); gifts from friends/relatives, 26.5 % (9/34), drug shops, 23.5 % (8/34) and health facilities, 26.5 % (9/34).

The reported importance of antimicrobial self-medication include, saving time [19, 25, 32, 35, 41–43, 45, 46], avoids crowding [23], and quick relief of the illness [35].

### Prevalence of antimicrobial self-medication

The prevalence of antimicrobial self-medication in low and middle income countries varied widely with some studies reporting as low as 4.0 % in Yemen [34] to as high as 91.4 % in Nigeria [46]. The overall estimate of antimicrobial self-medication in low and middle income countries is 38.8 % (95 % CI: 29.5-48.1). Reports from studies done in South America had a high, 44.1 % % (95 % CI: 9.7 %-78.6 %) overall prevalence of antimicrobial self-medication while the Middle East had the lowest 34.1 % (95 % CI: 23.4 %-44.9 %) (Table 3). The overall prevalence was higher among respondents who reported using multiple medicines, 61.9 % (95 % CI: 53.9 %-70.1 %), both antibacterial and antimalarial agents, 42.9 % (95 % CI: 19.6 %-66.3 %), antibacterial agents 33.4 % (95 % CI: 20.6 %-46.1 %) and lower in studies in which participants used only antimalarial agents only 30.3 % (95 % CI: 10.1 %-50.4 %). There was high heterogeneity in the included studies (I^2^ = 100 %) for all the sub-groups (geographic region, risk of bias, medicine used) assessed.

**Table 3:** Pooled results for the prevalence of self-medication by region, antibiotic used and condition treated

Category	Description	Number of studies (n = 34)	Number of respondents	Prevalence of SM	95 % CI
Geographic region	Sub-Saharan Africa	15	11667	40.6 %	25.8–55.8
	Asia	8	6980	38 %	15.2.60.8
	Middle East	8	11942	34.1 %	23.4–44.8
	South America	3	751	44.1 %	9.7–78.6
Risk of bias	Low	14	25009	39.2 %	21.6–56.9
	Moderate	12	3331	39.1 %	31.2–46.9
	High	8	1131	37.6 %	22.3–52.8
Medicine used	Antibacterial only	17	8486	33.4 %	20.6–46.1
	Antimalarial only	5	2411	30.3 %	10.1–50.4
	Antibacterial and Antimalarial	8	10818	42.9 %	19.6–66.8
	Multiple antimicrobial agents	4	735	61.9 %	53.9–70.1

SM: Self–medication, %: Percentage, CI: Confidence Interval

### Inappropriate practices of antimicrobial drug use in self-medication

The most common inappropriate practice in non-prescription use of antimicrobial agents include: short duration of treatment mostly less than five days [16, 17, 32, 44, 47], insufficient dose of medication [17, 19, 32, 47], wrong indication (use of antibacterial drugs in treating viral infections) [17, 22], and exchange/sharing of medicines [38, 40, 48, 49].

The use of antibacterial drugs in treating viral infections was mostly reported in studies done in the Middle East [22] and Asia [17]. The agents commonly used in treating symptoms of viral infections such as flu included; ampicillin, tetracycline, metronidazole, ceftriaxone, kanamycin, cotrimoxazole [17, 22]. Short duration of treatment (<5days) using antimicrobial self-medication was commonly reported in Asian studies, [16, 17]. Insufficient dose of medications used in self-medication was mostly reported in sub-Saharan African studies [19, 32] and Asia [17].

### Factors associated with antimicrobial self-medication in developing countries

The commonly reported factors that determined antimicrobial self-medication included; past successful use [33], low level of education [19, 21, 26, 33, 46], female gender [33], age [21, 33] and middle income [19, 21, 26, 33, 46]. Studies done in Africa reported; low level of education, severity of illness (mild-to-severe), female gender, age (≥45 years) and middle income as determinants of antimicrobial self-medication. Similarly in the Middle East, level of education, age (18-39 years) and middle income (Table 4).

**Table 4:** Factors that determine antimicrobial self-medication

Region	Number of studies	Studies with Multivariable regression analysis	Number of respondents	Determinants of antimicrobial Self-medication
Africa	3/15	Sapkota, 2010	706	Lower lever of education (OR: 2.8, 95 % CI: 1.1-7.1, P = 0.03)
				Non-science qualification (OR: 1.58, 95 % CI: 1.03-2.2.5, P = 0.04)
				Severity of illness (mild to moderate) (OR: 1.64, 95 % CI: 1.01-2.67, P = 0.05)
		Osemene, 2012	2000	Age (≥ 45 years) (OR: 3.4, P = 0.001)
				Female gender (OR: 3.8, P = 0.001)
		Awad, 2005	1750	Female gender (OR: 1.8, 95 % CI: 1.4-2.4
^a^Asia	0/8	None	None	None
Middle East	1/8	Al-Azzam, 2007	8864	Age (18-39 years) (OR: 1.59, 95 % CI: 1.3-1.95, P < 0.05)
				Education (primary) (OR: 2.1, 95 % CI: 1.09-2.08, P < 0.013)
				Income status (middle) (OR: 1.48, 95 % CI: 1.18-1.85, P = 0.001)
^a^S. America	0/3	None	None	None

^a^No single study done in South America or Asia performed multivariable regression analysis to establish determinants of antimicrobial self-medication

### Clinical outcomes of antimicrobial self-medication

The studies included in the review reported both positive and negative outcomes of the use of antimicrobial self-medication. The negative outcomes included; allergies [18, 22], lack of cure [23, 28, 37, 44] and causing death [22, 43]. While positive outcomes attributed to the use of antimicrobial self-medication included recovery from the illness [15, 28, 33, 35].

### Duration of use of antimicrobial drugs in self-medication

Twenty nine (29/34: 85.3 %) of the included studies did not report the duration which participants spent using antibiotics during an illness episode. In a study by Al-Azzam, 2007 [21], participants spent 4-7 days taking non-prescription antibacterial drugs during an illness. In studies by Deressa, 2003 and Sanjana, 2006 [23, 32] participants spent 1-3 days taking antimalarial drugs (Sulphadoxine-Pyrimethamine, chloroquine). Other studies [16, 17], reported participants spending less than five days taking non-prescription antibacterial drugs during an illness episode.

## Discussion

### Main findings

Community drug sellers often do not have adequate biomedical knowledge of the antimicrobial agents and the disease processes. However they are commonly used as source of advice or information for the antimicrobial agents obtained and used over-the-counter. Settings in which individuals are highly educated tend to have relatively low levels of use of antimicrobial self-medication. Therefore promotion of literacy among communities is an important target to minimize antimicrobial self-medication in LMICs. Due to their prior successful use of antimicrobial agents, individuals in most communities tend to believe that they are able to manage subsequent illness without consulting a physician. This is a potential risk factor for inappropriate drug use since most patients lack knowledge of the disease process and the medicines used in self-medication. In the review, adverse effects of antimicrobial self-medication were rarely reported in the articles from most studies in LMICs.

Responsible self-medication has the potential of being an important alternative to the formal healthcare system, providing patients the opportunity of accessing immediate healthcare [50]. However in most communities especially of developing countries, in addition to accessing medicines designated as over-the-counter, individuals also use prescription only medicines without any medical supervision. Such a practice is not likely to benefit patients especially in the case of antibiotics as it’s associated with potential risks to both the patient and community. The reason (s) why individuals decide to use medicines designated as prescription only without any guidance from a health professional are unique to different settings and are reflective of a matrix of health system, societal, economic and health factors [11]. Therefore establishing these factors is a critical step in designing and implementation of interventions against irresponsible self-medication. The current review also presents estimate of the prevalence of antibiotic self-medication and the associated clinical outcomes in communities of LMICs.

In this review, the prevalence of antimicrobial self-medication in LMICs was 38.8 % and is consistent with the findings (39 %) of a previous review on global antimicrobial self-medication [3]. The use of antibiotics without a prescription occurs globally despite their prescription only legal status in most countries [3]. Our review revealed that the prevalence of non-prescription antibiotic use in LMICs is similar to the global rate. However the high levels of poverty as individuals cannot afford full antibiotic course and illiteracy potentially increase the likelihood of risks associated with non-prescription antibiotic use in LMICs [19]. For example development of antibiotic resistance, a consequence of inappropriate drug use commonly associated with self-medication causes higher mortality in LMICs compared to the developed nations [51]. Our review also showed that the prevalence of antibiotic self-medication varied in different regions. This could be due to the difference in the effectiveness of enforcement of regulations on antibiotic self-medication in different resource limited countries. However, there was significant heterogeneity in the outcome of studies included in the review even after we performed sub-group analysis (region and risk of bias). Therefore we could not combine the included studies in a meta-analysis.

Self-medication has potential benefits which are shared among patients, healthcare professionals, healthcare system, and the pharmaceutical industry. For the industry; increased access to the products results in more profits; health professionals avoid unnecessary consultations with patients having minor symptoms; healthcare costs to government are reduced as individuals meet their healthcare bills and patients gain greater empowerment thus improving patient-clinician relationship [10]. A study included in this review reported rapid resolution of disease symptoms among participants [52]. Others showed that using antimicrobial self-medication, saves time, is affordable, and convenient. These positive attributes of self-medication, further reinforce community use of antimicrobial self-medication in management of prevalent illnesses. However, it should be noted that the potential benefits associated with self-medication will only be achieved if it’s done responsibly and the medicines used are safe, efficacious and information leading to their safe use is easily accessible to the communities [11].

The underlying challenges of health systems in most LMICs such as inadequate healthcare potentially influence use of self-medication [11]. In addition, the lack of policies or their inadequate implementation enables easy over-the-counter access of antibiotics [53]. A previous study in northern Uganda found that over half (59.3 %) of community members who practiced antimicrobial self-medication were not aware of any restrictions on their non-prescription use in the country [54]. This occurs in spite of the existence of national drug policy formulated in 2002 which limits antibiotics to prescription only use. Furthermore, most LMICs face the challenge of irregular supply of drugs to the public health facilities which limits community access to healthcare. This coupled with the high burden of infectious diseases in these countries makes the private sector an important alternative source of healthcare [6]. However, the profit oriented nature of service delivery in this sector in addition to the inadequate supervision, influence over-the-counter sale of antibiotics despite their prescription only legal status. The question facing most LMICs who suffer high burden of infectious diseases is how to balance improved access to antibiotics for individuals with true infectious diseases through self-medication while at the same time ensuring appropriate use.

The key determinants of antimicrobial self-medication in LMICs included; severity of illness, economic status, past successful use and educational level. Most community members do not visit a health professional prior to initiating treatment due to the associated costs such as time, travel expenses and consultation charges [55]. The high level of poverty in communities of most LMICs in addition to the fact that patients can purchase antibiotics over-the-counter using any amount of money influence antibiotic use practices [11]. The prevalent belief in self-efficacy among patients due to past illness experiences further impacts on the use of antibiotic self-medication. Community members with a high level of education were more likely to use antimicrobial self-medicate possibly due to the exposure and increased awareness on health [56]. Patients who assessed the symptoms of their illnesses as mild or moderate were more likely not to consult a healthcare professional. However, lack of biomedical knowledge of the disease symptoms is likely to increase the risk of inappropriate antibiotic use practices such as stopping treatment when symptoms resolve [11], delay in seeking appropriate treatment which may potentially result in more severe disease. Delay in seeking medical care has been associated with increased mortality among patients suffering from treatable infections such as malaria [57]. The decision by individuals in communities to use self-medication is as a result of complex interaction of various factors such as quality of healthcare, regulatory environment, burden of disease, economic factors and belief [11]. Therefore interventions to mitigate antibiotic self-medication especially in LMICs need to specifically focus on these primary factors.

The review established inappropriate practices in antibiotic self-medication in communities of LMICs. These included, not completing dose, sharing drugs, stopping use of drugs when symptoms improve and inaccurate indication. The use of antibacterial agents in treatment of common cold was reported in studies done in Jordan [21], Iran [24], Palestine [30], Vietnam [43], and Sudan [19]. Not completing the dose of antibiotics carries a risk of clinical failure. Previous studies done in children with mild pneumonia using 3 day and 5 day amoxicillin found non-compliance as the main reason for treatment failure [58, 59]. The majority of studies included in the review reported self-medication using multiple antimicrobial agents. The use of more than one antibiotic during an illness episode is indicative of the uncertainty of the cause of illness. These inappropriate practices potentially increase the risk of mistreatment, adverse drug reactions, resistance development and drug interactions [6, 7, 10]. This is further worsened by the high burden of infectious diseases in addition to the limited therapeutic choices in most LMICs [6]. Antibiotic resistance is likely to add further financial strain to the healthcare system which is already faced with the challenge of inadequate funding. This is especially the case as patients with resistant infections are likely to stay longer in hospitals and the need to use more expensive second line antibiotic drugs. Agencies such as World Health Organization (WHO), World Self-medication Industry (WSMI) and the ministries of health of LMICs need to establish specific interventions focusing on these common inappropriate antibiotic use practices.

The review found that drug sellers, previous successful use, drug leaflets, past prescriptions and friends or relatives were the main sources of drug information in self-medication. Drug leaflets are an important source of information, however poor readability makes using them challenging to use [6]. In addition, the high level of illiteracy in LMICs further limits the effectiveness of leaflets as a source of information. Providing this information in indigenous languages in addition to well written information could improve the usefulness of drug leaflets in these settings [6]. Prescription practices of physicians in communities are likely to influence antibiotic use behavior of the local population as patients commonly refer to old prescriptions in choosing medicines used in self-medication [11]. Interventions such as retention of prescriptions in the pharmacy could help mitigate use of old prescriptions in making treatment decisions in communities [60]. Drug sellers in most of the developing countries have less impetuous of knowing biomedical information of the drugs they sell as they assume that patients know what they want and for them they know the price [50]. In Lao People’s Republic, over half (59 %) of drug dispensers are not knowledgeable about the drugs that they were selling [61]. However, in this review drug sellers were frequently reported as a source of information for antibiotics obtained over-the-counter despite their potential lack of biomedical knowledge of these drugs. This increases the risk of misinforming their clients on the antibiotic agents accessed over-the-counter. Non-prescription use of antibiotics without relevant information on how to take them, indications, adverse effects and contraindications could potentially expose patients to the risk of inappropriate drug use [62]. Health personnel in communities of LMICs are thus an important target for sensitization, monitoring and support supervision.

The review had some limitations, variation in the techniques of data collection and reporting in the primary studies which were included. There was a potential of bias in the included studies due to; method of analysis, recall, selection and social desirability. This invariably has an effect on the findings of the primary studies. For example, majority of studies used recall period of more than six months while others did not report the duration of recall used during data collection. In a previous study [63], it was found that a recall period of more than one month was significantly associated with the risk of recall bias. The use of non-random methods in participant recruitment in addition to not validating the data collection tools was common in most surveys and could potentially have an effect on the study outcomes. There was high heterogeneity in the studies reviewed possibly due to lack of standardized criteria of survey data collection. We were unable to access some articles during the study selection in spite of all the efforts taken. The studies included in the review rarely reported on the negative outcomes of antibiotic self-medication experienced by community members. This could be due to limited knowledge of the antibiotic medicines that they used in self-medication [34].

## Conclusions

Antimicrobial self-medication is highly prevalent in resource limited countries and is commonly associated with inappropriate use. Although self-medication is an important alternative to the formal health sector especially in most LMICs, it is imperative that decisions to use non-prescription antimicrobial agents are both safe and appropriate if the potential benefits are to be maximized with minimal risks. Educational interventions targeting both health personnel and community members in addition to improving access to quality of public healthcare, enforcement of regulations on non-prescription medicine use, and reducing the burden of infectious diseases could help mitigate the challenge of non-prescription antibiotic use in LMICs. The practice of referring to old prescriptions and past successful treatment experiences by the communities is key areas of focus for the interventions. There is an urgent need to development and validate a method for collecting data on community antimicrobial use to help improve the quality of evidence from such survey studies.

## Additional file

Additional file 1:
**Appendix 1.** Table S5: Risk of bias assessment of included studies.
