# Supplementary material for: Household antimicrobial self-medication: a systematic review and meta-analysis of the burden, risk factors and outcomes in developing countries
Source: BMC Public Health. 2015 Aug 1;15:742. doi: 10.1186/s12889-015-2109-3 (PMC4522083; doi:10.1186/s12889-015-2109-3)
Supplement: Additional file 1: — Appendix 1. Table S5: Risk of bias assessment of included studies. [file 12889_2015_2109_MOESM1_ESM.doc]

|  | Selection bias due to sampling | Selection bias due to proportion of responders (>59.9%) | Selection bias due to baseline characteristics (& confounding) | Detection bias due to recall period (≤ 1 month) | Detection bias due to reliability of measurement tools used | Detection bias due to social desirability | Bias due to method of data analysis used for overall outcome | Bias due to method of data analysis used for associated factors |  |
| --- | --- | --- | --- | --- | --- | --- | --- | --- | --- |
| Agbor, 2011 | – | – | – | ? | – | – | – | + | Low |
| Abdo-Rabbo, 2003 | + | – | ? | ? | – | + | – | + | High |
| Al-Azzam, 2007 | + | – | – | – | – | – | – | + | Low |
| Askarian, 2012 | – | – | – | + | – | – | – | + | Low |
| Auta, 2012 | – | + | + | – | – | ? | + | ? | High |
| Awad, 2005 | – | – | – | – | – | – | – | – | Low |
| Bano, 2012 | ? | ? | + | ? | – | – | + | + | High |
| Barah, 2010 | – | – | – | – | – | ? | – | + | Low |
| Chowdhury, 2009 | – | + | – | + | ? | – | – | + | Moderate |
| De Oliveira, 2004 | ? | – | – | + | – | ? | + | + | High |
| Deressa, 2003 | – | – | + | + | ? | + | – | – | Moderate |
| Enato, 2010 | – | – | ? | – | – | – | – | + | Low |
| Hussain, 2011 | – | – | – | – | ? | – | – | + | Low |
| Jassim, 2010 | ? | – | – | ? | – | ? | – | + | Moderate |
| Jombo, 2011 | – | – | + | ? | – | ? | – | + | Moderate |
| Lima, 2009 | + | ? | ? | ? | + | + | + | + | High |
| Mossa, 2012 | – | – | – | + | – | ? | – | + | Moderate |
| Nounon, 2009 | ? | ? | – | ? | + | – | + | + | High |
| Ngasha, 2011 | + | – | + | ? | ? | – | – | + | High |
| Okumura, 2012 | – | – | + | – | – | – | – | – | Low |
| Omole, 2010 | ? | – | + | + | ? | – | – | + | High |
| Onanuga, 2011 | – | – | – | ? | + | ? | – | + | Moderate |
| Onohwosafe, 2013 | – | – | – | ? | – | – | – | + | Moderate |
| Osemene, 2012 | + | – | – | ? | – | – | – | – | Low |
| Oyetunde, 2010 | + | – | ? | ? | – | ? | – | – | Moderate |
| Sanjana, 2006 | – | – | – | ? | – | – | – | ? | Low |
| Sapkota, 2010 | – | – | + | + | – | – | – | – | Low |
| Sarahroodi, 2009 | – | – | + | + | – | – | – | + | Moderate |
| Shankar, 2002 | – | – | + | + | ? | ? | – | – | Moderate |
| Shehadeh, 2012 | – | – | + | ? | – | ? | – | + | Moderate |
| Sawalha, 2008 | – | – | – | ? | + | – | – | + | Low |
| Sihavong, 2006 | – | – | + | – | – | – | – | + | Low |
| Widayati, 2011 | – | – | – | – | – | – | – | ? | Low |
| Yousif, 2002 | ? | – | ? | ? | – | – | – | + | Moderate |

**Appendix 1: Table 5: Risk of bias assessment of included studies**

**Symbols used mean Low risk (–), High risk (+), Not clear (?). Risk of bias category “not clear”, was combined with “high risk” for the aggregate assessment*
